# Osseous Metaplasia Mimicking Long Bone in Intramuscular Vascular Malformation

**Published:** 2018-05

**Authors:** Ritesh Panda, Mahesh Mangal, Sasidhar Reddy, Anubhav Gupta, Swaroop Singh Gambhir, Bheem Singh Nanda

**Affiliations:** Sir Gangaram Hospital, New Delhi, India

**Keywords:** Vascular malformation, Ossification, Equinus deformity foot, Osseous metaplasia

## Abstract

We describe a case of intramuscular vascular malformation with extensive ossification within the posterior compartment muscles of leg which grossly mimicked long bone leading to equinus deformity of foot. Phleboliths and calcifications are characteristic of vascular malformations, while massive ossification is rare. To our knowledge, no extensive ossified vascular malformation within the leg muscle has been reported. Intramuscular vascular lesions occur with an incidence of 0.8%, most frequent in upper and lower extremities. Most are venous malformations. The diagnosis is rarely made before surgery and requires a definitive histological analysis, as there are no pathognomonic clinical or radiographic findings, especially with extensive ossification. Spontaneous regression does not occur, and excision of lesion is due to aesthetic and functional disturbances.

## INTRODUCTION

Vascular anomalies are diverse and represent a broad spectrum of lesions of multiple pathologies that occur at many anatomic locations. It is a new and rapidly developing discipline that demands teamwork between various specialties. For centuries, vascular birth marks were called by vernacular names derived from folk beliefs. It was believed that these were imprinted on the unborn child by a mother’s emotions or diet. Adjectives such as “cherry,” “strawberry” and “port-wine” have their roots in these traditional beliefs.^1^

A patient with vascular anomalies becomes a wanderer because of the improper diagnosis, complexity of treatment, limited outcome and lack of expertise available with individual doctors. Introduction of a biological classification system^2^ in 1982 cleared the terminological confusion of vascular anomalies and was accepted by the International Society for Vascular Anomalies in 1996. This scheme evolved from studies that correlated physical findings, natural history, and cellular features.^3^ There are two major categories of vascular anomalies, tumors and malformations. Vascular tumors are endothelial neoplasm characterized by increased endothelial turnover; malformations are the result of abnormal development of vascular elements during embryogenesis. Calcification within vascular malformation is a common finding; but ossification within the malformation of the extremities is rarely reported.^4^^-^^8^

Surgical excision is the treatment of choice for symptomatic lesions.^9^ While ossified intramuscular vascular lesions have been reported in the extremities,^10^ to the best of our knowledge, ossified intramuscular vascular malformation resulting in painless equinus deformity of ankle joint has not been previously reported in the literature. The clinical, radiologic, magnetic resonance angiographic and histological features are discussed here. We believe that massive ossification is a rare phenomenon in vascular lesions and deserves recognition because it can pose diagnostic challenges.

## CASE REPORT

A previously healthy 42 years old male presented to our institute with history of gradually progressive and painless swelling over left calf since two months. He was a non-smoker, laborer by occupation. The patient noticed a firm swelling in calf region of left leg 5 years ago. No history of trauma or infection prior to the appearance of the mass was reported. No family history of any such swelling in the past. Patient was operated for swelling over calf region 20 years back but no records were available. There was a history of gradual increase in deformity of left foot since 3 years and the patient had started walking on toes on left side with no dorsiflexion at ankle joint. 

**Fig. 1:**
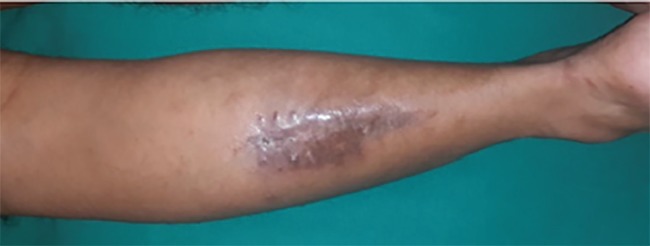
: Preoperative picture showing swelling and scar over left calf with equines deformity of foot

**Fig. 2:**
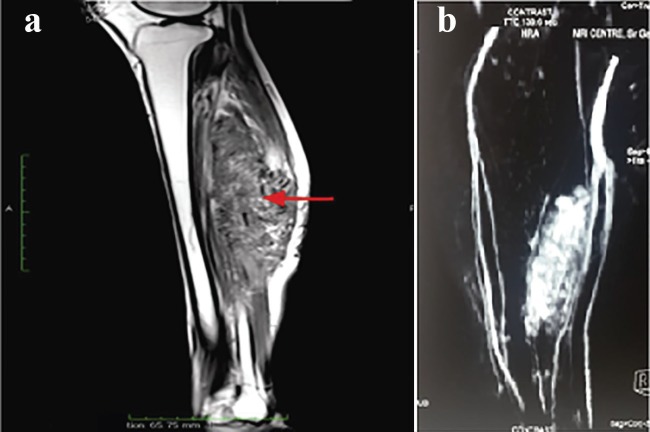
**a:** MRI showing arterio-venous malformation in muscle compartment. **b:** MR Angiography showing ossification within vascular malformation.

On examination, there was a single, non-tender, hyperpigmented scar of size 8×3 cm over mid-calf region fixed to underlying structures. A large, well defined non-tender, firm, swelling was palpable in posterior aspect of left leg measuring about 28×8 cm extending from tendoachilles region up to 5 cm distal to popliteal fossa and medially and laterally up to border of tibia and fibula respectively. The overlying skin was normal with no discoloration and local raise of temperature. Movement of knee joint was normal. There was fixed equinus deformity of left foot (Figure 1). No inflammatory signs, skin changes or adenopathies were present. No bruits were heard on auscultation. Neurovascular examination of left leg and foot was normal. Laboratory findings were within normal limits. Radiological examination revealed large soft tissue mass with linear and streak-like ossification around the left tibia. MR Angiography (Figure 2a, 2b) showed arteriovenous malformation in left calf with multiple feeding arteries arising from popliteal, peroneal and anterior tibial artery and large draining veins draining deep into venous system of leg. The tibia and fibula marrow showed normal signal intensity.

**Fig. 3:**
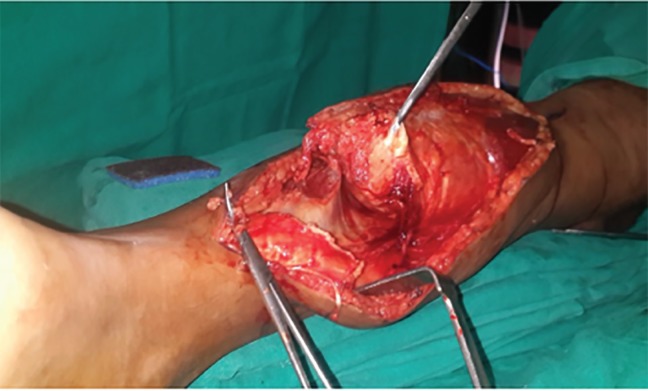
Intraoperatively tumor seen involving superficial compartment muscles of leg.

Because of patient symptoms and with clinical diagnosis of a vascular malformation, a wide surgical excision of the lesion was done. Through a 25 cm longitudinal incision across the calf, posterior compartment muscles were exposed. The mass was found completely involving superficial group of posterior compartment muscles sparing the deep compartment with no attachment to periosteum or bone (Figure 3). Peroneal artery and vein were found to be embedded in the lesion and thus sacrificed. Plane of dissection was between superficial and deep muscles.

**Fig. 4:**
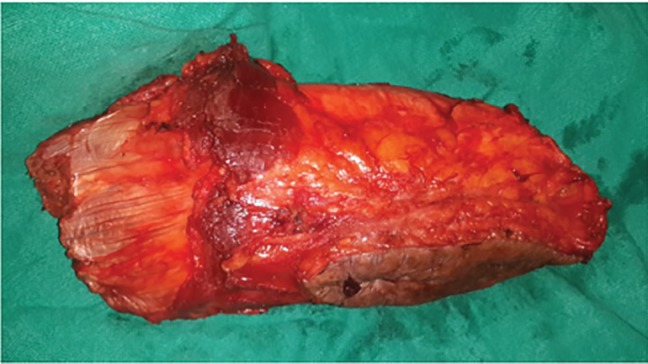
Excised lesion with attached muscles and scar. Stony hard feel on touch

**Fig. 5:**
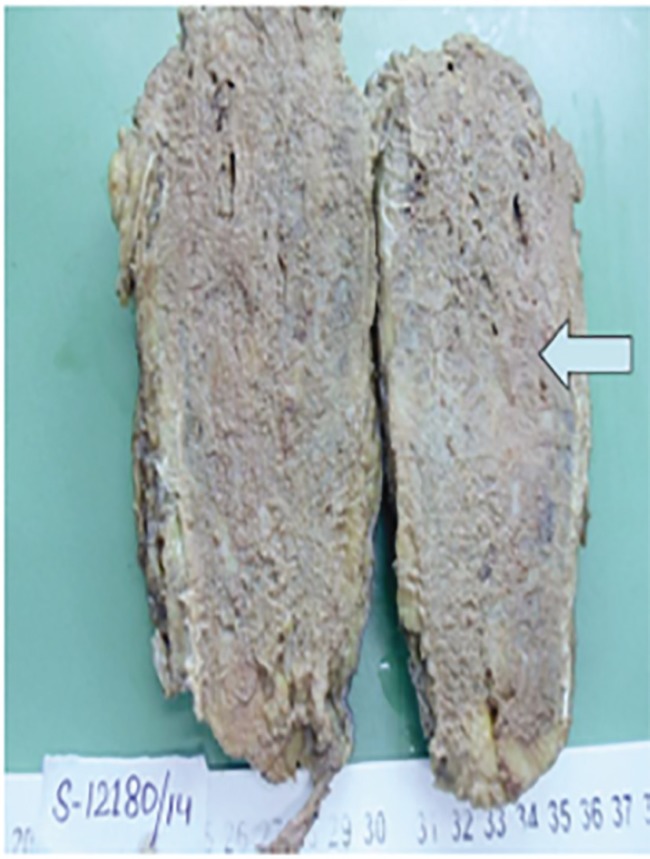
Gross: segment of ossified tissue which is grey white, gritty and congested

The lesion was completely removed along with overlying cutaneous scar with wide surgical margins leaving posterior tibial artery in continuity. Intraoperative, complete dorsiflexion of foot was achieved with intact vascularity of leg. The excised specimen was very hard like bone and had to be cut longitudinally with saw (Figure 4). Grossly the resected specimen showed ossified tissue covered with skin and soft tissues including muscle, tendons and adipose tissue measuring 15×7×5 cm. The cut surface of the ossified area was grey white, gritty and congested (Figure 5). Microscopically, it revealed features of a vascular malformation with numerous blood vessels of variable size and shape composed of arteries and veins which were dissecting soft tissues and interstitial planes of skeletal muscle. 

**Fig. 6:**
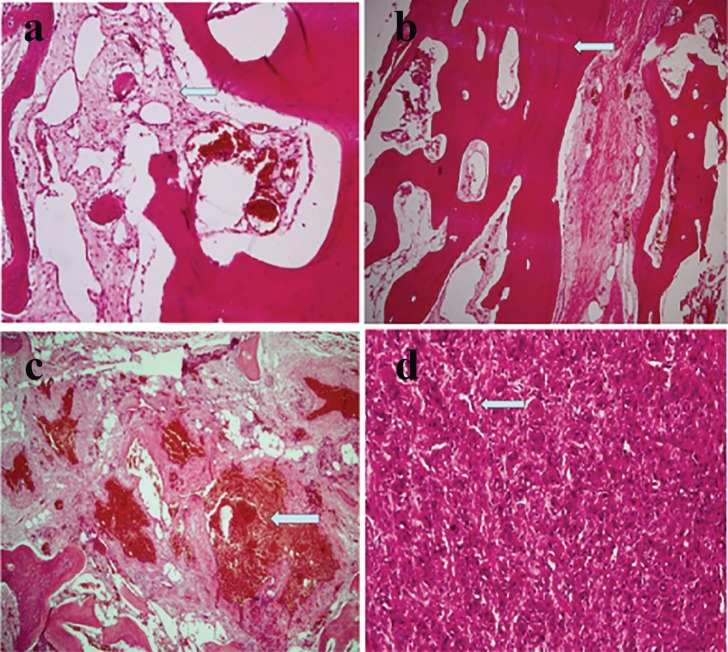
**a:** Numerous blood vessels of variable size and shapes surrounded by bony trabeculae. **b: **Formation of mature lamellar bone in the malformation. **c:** Some of the vessels showing fresh and organized thrombi. **d:** Focal areas showing numerous proliferating capillaries with plump endothelial cells.

Many of the vessels were thin walled with anastomosing and a sinusoidal appearance. Some of them showed fresh and organized thrombi within this vascular background, extensive osseous metaplasia characterized by mature lamellar bone formation was seen. (Figure 6a-d). The diagnosis was consistent with arterio-venous malformation with extensive osseous metaplasia. At the time of recent follow up after one year from the operation, no local recurrence of the tumor was demonstrated clinically and radiologically. No restriction of motion of ankle joint was found. Patient is presently walking with a normal gait.

## DISCUSSION

Intramuscular vascular lesions have an incidence of 0.8%.^11^ The first description of a case of intramuscular hemangioma is attributed to Liston in 1843, which called this as “erectile tumor”.^12^ They are most frequent in the upper and lower extremities and trunk, occurring in the head and neck region in 15% of cases.^12^ Intramuscular hemangiomas can be either asymptomatic or symptomatic including pain, increased girth of the extremity or swelling and increased temperature or discoloration of the overlying skin. A palpable mass is seen in the vast majority of the cases. Larger hemangiomas may be associated with a bruit or thrill. It usually occurs before the age of 30 with equal sex distribution.^13^ The aetiology of intramuscular haemangioma still remains unknown.^14^ In addition to their vascular components, vascular malformations can show calcification, fat, smooth muscle and fibrous tissue, with fat being the most common component. Calcification and ischemic changes are rare, but recognized in venous malformation in brain (also referred to as venous angiomas or developmental venous anomalies) which are best appreciated on contrast enhanced MR.^15^^,^^16^

Most intramuscular vascular lesions described in the literature as haemangiomas are not haemangiomas but venous malformations.^11^ Few cases have been reported of an extensively ossified venous malformation within the mylohyoid muscle^11^ and thigh.^13^^,^^17^^,^^18^ Ossification in the skeletal muscle vascular lesions is a rare phenomenon and less commonly described. The first case was reported as an orange-sized swelling in the right posterior lower leg of a 19 year old patient by Bishop in 1963.^13^

Differential diagnoses of ossified intramuscular vascular malformation radiologically include myositis ossificans, ossifying fibromyxoid tumor, fibrodysplasia ossificans, and extra skeletal osteosarcoma.^13^ X-Rays of ossified intramuscular hemangiomas showed irregularly ossified lesions in the soft tissues, the so-called “Swiss cheese” appearance described by Engelstadet *et al.*^19^ This appearance reflects the architecture of the mature bone interspersed with large cavernous vascular channels. Unlike appearance described by Engelstad *et al.*, Swiss-cheese-like appearance was not detected in our case but linear and streak-like calcification was seen radiologically in our case.

Asymptomatic intramuscular vascular malformation is observed closely. Indications for surgery include persistent pain, progressive increase in size of the mass, functional impairment and patient anxiety.^13^ The location and extent of the lesion must be determined for treatment planning and surgical resection remains the treatment of choice. Complete surgical excision with a surrounding margin of normal muscle is mandatory, using a minimally traumatic surgical technique and coagulation of all of the feeding vessels. Local recurrence rate could be up to 50%.^17^

Malignant transformation is rare. Lesions arising in muscle may create significant diagnostic uncertainty, as on clinical examination they are little different from soft tissue neoplasms and often imaging features may not be fully appreciated or easy to interpret. Skeletal muscle vascular malformations are uncommon soft tissue tumors that are completely treatable, the knowledge of their natural history, clinical findings, and imaging appearances are of great importance for proper diagnosis. Although rare, awareness of extensive osseous metaplasia is important for establishing diagnosis in view of its benign nature and also in excluding it from other bone forming lesions. To summarise, we are reporting a case of ossified intramuscular vascular malformation with its peculiar clinical, radiologic and histological features which shows a rare presentation of a relatively common tumor.

The aim of reporting this case is to emphasize that, in cases with chronic or recurrent painful or painless swelling in a muscle or adjacent to a joint, associated with loss of function and deformity with or without an episode of trauma should alert the surgeon to this diagnosis. Investigations such as ultrasound can diagnose the lesion but may not be able to delineate its extent; MRI is preferred for identifying the exact location, extent, and size of the lesion for planning surgical excision, which isthe most preferred treatment for these lesions. Histological diagnosis is confirmatory. Patient should also be counselled for the possibility of local recurrence before surgery.

## CONFLICT OF INTEREST

The authors declare no conflict of interest.
